# Cold-water coral mortality under ocean warming is associated with pathogenic bacteria

**DOI:** 10.1186/s40793-024-00622-0

**Published:** 2024-10-16

**Authors:** Mathilde Chemel, Erwan Peru, Mohammad Binsarhan, Ramiro Logares, Franck Lartaud, Pierre E. Galand

**Affiliations:** 1 Sorbonne Université, CNRS, Laboratoire d’Ecogéochimie des Environnements Benthiques (LECOB), Observatoire Océanologique de Banyuls, F-66650 Banyuls-sur-Mer, France; 2 Institute of Marine Sciences (ICM), CSIC, Barcelona, Spain

**Keywords:** Deep-sea corals, *Lophelia pertusa*, North Atlantic Ocean, Pathogens, Holobiont, Metagenome, Climate change

## Abstract

Cold-water corals form vast reefs that are highly valuable habitats for diverse deep-sea communities. However, as the deep ocean is warming, it is essential to assess the resilience of cold-water corals to future conditions. The effects of elevated temperatures on the cold-water coral *Lophelia pertusa* (now named *Desmophyllum pertusum*) from the north-east Atlantic Ocean were experimentally investigated at the holobiont level, the coral host, and its microbiome. We show that at temperature increases of + 3 and + 5 °C, *L. pertusa* exhibits significant mortality concomitant with changes in its microbiome composition. In addition, a metagenomic approach revealed the presence of gene markers for bacterial virulence factors suggesting that coral death was due to infection by pathogenic bacteria. Interestingly, different coral colonies had different survival rates and, colony-specific microbiome signatures, indicating strong colony-specific variability in their response to warming waters. These results suggest that *L. pertusa* can only survive a long-term temperature increase of < 3 °C. Therefore, regional variations in deep-sea temperature increase should be considered in future estimates of the global distribution of cold-water corals.

**Supplementary Information:**

The online version contains supplementary material available at 10.1186/s40793-024-00622-0.

## Introduction

In the deep sea, cold-water corals form extensive reefs that represent highly valuable habitats, providing nursery grounds, and a source of food for numerous marine species [12]. These key ecosystem engineers thus act as local biodiversity enhancers and provide many ecosystem and ecological services [3]. Nevertheless, these corals now face serious anthropogenic threats, particularly in submarine canyons, due to unsustainable fishing activities, pollution, and global warming [54]. Sea water is warming down to the deep ocean [5], and projected climate scenarios indicate that warming is likely to be faster in deep environments than at the surface of the oceans in the future [9]. In the Atlantic Ocean, mesopelagic and bathypelagic water temperatures are predicted to rise by up to 3 °C before the end of the century [72].

Temperature changes are known to impact the health of cold-water corals by changing their associated microbiome (i.e., associated microbial communities), organic carbon content, respiration, feeding behavior and skeletal biomineralization, which could, in some cases, lead to coral death [10, 19, 22, 30, 59]. In the Mediterranean Sea, where corals live at 13–14 °C, a recent study showed that *Lophelia pertusa* (now synonymized as *Desmophyllum pertusum*, [1]), the main reef-building and most widespread cold-water coral species, had reduced physiological functions when exposed to warmer waters. It also appeared to be less tolerant to warming than *Madrepora oculata*, another common coral of the deep-sea reefs [19]. The study conducted at the holobiont level, considering both the coral host and its microbiome, showed that at warmer temperatures, physiological activity, growth rate, and energy reserves were reduced, and behavior was altered with increased polyp activity. At the same time, the coral microbiome changed in composition, with the appearance of potential opportunistic bacteria [19].

The coral microbiome has been shown to be closely linked to the health and growth of tropical corals as it contributes significantly to the host metabolism [8], and may contribute to stress tolerance [6]. However, while some microorganisms are beneficial to their host, others can cause coral diseases [66, 78]. The microbial communities may, therefore, be a good indicator of tropical coral health [29]. In the case of cold-water corals, a number of studies have shown that the microbiome is affected in its composition and diversity in response to environmental changes [17, 19, 25, 26, 33, 48]. Although the role of the associated bacteria and their potential metabolisms have begun to be investigated [34, 52, 64], the extent of the potential functions of cold-water coral microbiome remains largely unknown.

In the north-east Atlantic Ocean, cold-water coral populations live at lower temperatures than those in the Mediterranean Sea (i.e., 8–12 °C in the Gulf of Biscay [76]). As deep-sea temperatures are rising, it is not yet known whether *L. pertusa* in the Atlantic are already living at their thermal optimum, making them highly vulnerable to global warming, or whether they can thrive in waters as warm as their Mediterranean counterparts, and would therefore be less affected by future thermal changes. Knowledge on the impacts of warming on Atlantic *L. pertusa* remains limited to respiratory physiology [22, 31] and growth [13].

In this context, the aim of the present study was to determine, under laboratory conditions, the effects of elevated temperatures on the Atlantic reef-forming cold-water coral *Lophelia pertusa*. A two-month aquaria experiment was conducted in which corals from a submarine canyon in the Bay of Biscay (north-east Atlantic Ocean) were exposed to three different temperature conditions: the in situ temperature (10 °C), and temperatures corresponding to two different warming scenarios (13 °C and 15 °C). The coral response was investigated at the holobiont level by measuring coral survival and growth, and by describing the diversity of the microbiome by metabarcoding, and its functions using metagenomics.

## Material and methods

### Specimen collection and maintenance

Corals were sampled in the Lampaul canyon in the Bay of Biscay, North-east Atlantic Ocean (47° 36.703 N, 07° 32.192 W). The Lampaul canyon has been included in a newly defined Natura 2000 area, with the aim of preserving deep-sea reef habitats. As early as in the 1950s, Le Danois [42] described the reef as a particularly rich coral area. Recent dives have revealed a great diversity of coral habitats in this area although severely impacted by fishing activities [76]. Five distinct colonies of *L. pertusa* (Linnaeus 1758) (orange specimens) were collected at 800 m depth, within a water mass corresponding to the Mediterranean Outflow Water [21]. Samples were collected using the remotely operated vehicle (ROV) Ariane from the R/V Thalassa, during the research cruise ChEReef (habitat Characterization and Ecology of cold-water coral Reefs) in August 2021 [49]. On board, the corals were maintained in oxygenated seawater at the ambient seabed temperature of 10 °C, using a cooling unit (ICE400, Aquavie, Connaux, France). Once at the laboratory (Banyuls Oceanological Observatory), the colonies were maintained for 5 months at their in situ temperature to acclimatize to laboratory conditions in a dark, thermoregulated room, in aerated 80 L tanks, continuously supplied (> 1 renewal day^−1^) with filtered (5 µm) seawater pumped from 10 m depth. Corals were fed three times a week alternately with freshly hatched *Artemia salina nauplii* (350 L^−1^) and 5 mL of marine snow plankton diet (Two Little Fishies Inc, Miami Gardens, Florida, USA), to provide a complete and diverse nutrient supply [26]. After the acclimatization period, each of the *L. pertusa* colonies were cut into small fragments, hereafter referred to as nubbins, each composed of 3–13 living polyps [62]. The cutting technique does not harm the nubbin [62]. The nubbins were glued onto PVC blocks using an aquatic epoxy resin (Hold Fast Sand, Aquarium System, Sarrebourg, France), and then transferred to experimental tanks.

### Experimental design

*Lophelia pertusa* nubbins were exposed to three different temperature conditions: 10, 13 and 15 °C. The 10 °C condition represented the in situ temperature (control), 13 °C (+ 3 °C increase) corresponded to the in situ temperature in the Mediterranean Sea, and 15 °C (+ 5 °C increase) represented a severe warming near the presumed upper limit of thermal tolerance for this species [10]. Experiments were conducted following the protocol detailed in Chapron et al. [19]. Nubbins (i.e., subsamples of colonies) from four different colonies were present in each experimental condition. Each colony was represented by 6–7 nubbins randomly distributed in each of the three 36L experimental tanks, with sufficient distance between the nubbins to avoid any contact between the different nubbins and their polyps [62]. Five nubbins from each colony, each composed of at least 3 polyps, were dedicated to microbiome analysis. One to two nubbins from each colony, each composed of at least 5 polyps, were allocated for growth measurements (Supplementary Fig. 1).

Each experimental tank was equipped with a small water pump (NJ400, Newa Jet, Loreggia, Italy) that maintained a constant flow of 3 cm s^−1^ to facilitate water mixing. To ensure stable temperature regulation, each experimental tank was placed in a larger water bath tank, and equipped with a temperature probe connected to a temperature controller (Biotherm Eco, Hobby Aquaristik, Gelsdor, Germany, precise at 0.1 °C), which was coupled to the water bath tank’s cooling unit (ICE400, Aquavie, Connaux, France). The temperature in each tank was monitored every 30 min using an autonomous IBUTTON probe and manually checked twice a day using a digital thermometer (Checktemp thermometer, Hanna Instrument, Woonsocket, USA). The pH, oxygen concentrations, and salinity were measured manually twice a week (Supplementary Table 1), using probes (C3010 Multi-parameter analysers, Consort). Nubbins were acclimated for two weeks in the experimental aquaria prior to temperature changes. Then, the water temperature of the experimental tanks was gradually increased to avoid thermal shock. Temperature was raised over 10 days until the targeted temperatures were reached, following the protocol described by Naumann et al. [59]. The feeding routine was maintained throughout the experiment.

### Coral survival and skeletal growth

Throughout the experiment, polyps of each nubbin were thoroughly checked and noted as either dead or alive to assess survival rate. Survival rate was assessed at the polyp level as the percentage of surviving polyps at each sampling time relative to the initial number of polyps for each temperature condition.

**Fig. 1:**
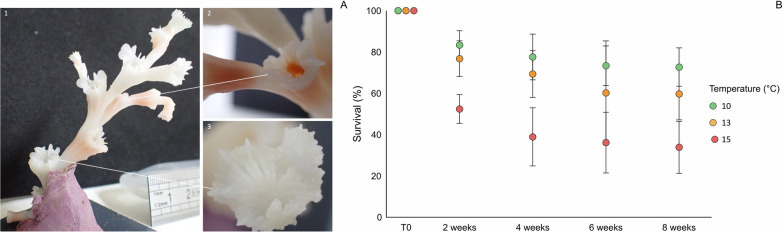
Photo showing a *L. pertusa* nubbin (1) with living polyps characterized by a pink colour (2) and white dead polyps (3) (**A**). Polyp survival rate (mean values from four colony replicates *per* time point and error type are presented) of *L. pertusa* at 10 °C, 13 °C and 15 °C at T0 (n = 120, 122, 124 polyps respectively at 10, 13 and 15 °C), after 2 weeks (n = 100, 94, 64 polyps), 4 weeks (n = 93, 85, 48 polyps), 6 weeks (88, 73, 45 polyps) and 8 weeks (87, 72, 42 polyps) (**B**)

To determine if polyps were alive, their colour was first observed. Healthy polyps from the Lampaul canyon are pink/orange, and a change of color inside the calyx was considered a strong indicator of polyp death (Fig. 1a). In cases of doubt, polyps were rinsed with seawater and deemed dead if there were no remaining tissues or if the tissues had detached from the calyx. When all the polyps of a nubbin were dead, the nubbin was removed from the aquarium.

Skeletal growth was measured using fluorescent calcein staining and by measuring polyp linear extension [39], as well as by a novel protocol of structured-light 3D scanning. At the beginning of the experiment, 1–2 nubbins (> 5 pol) *per* colony, dedicated for growth monitoring were stained with calcein fluorescein at 150 mg L^−1^ following the protocol described by Chapron et al. [16]. They were then scanned using the automatic AutoScan Inspec (Shining 3D, China) before starting the experiment (Supplementary Fig. 2). More precisely, nubbins were scanned by taking successive photos of the nubbin in several layers. The Ultrascan software aligned and superimposed these photos to form a cloud of points, which was then smoothed to reconstruct the nubbin in 3D. The software computes the volume of the 3D image hereafter called biovolume.

After two months, the nubbins were scanned again, using the same protocol as at the start of the experiment. They were then cleaned in a hydrogen peroxide solution (H_2_O_2_, 4%) at 60 °C for 12 h to remove all organic tissues, and rinsed with demineralised water. Then, each polyp calyx was individualised, placed on a slide, and glued with Patafix© in order to identify polyp’s septa under a microscope. The calcein labelling was observed under a fluorescence microscope (Olympus IX51, Olympus, Tokyo, Japan) with excitation at 495 nm. Images were captured with a camera and image analysis was carried out using Image J software. Growth was assessed by measuring the distance between the calcein label and the outer edge of the septum of the calyx (repeated 10 times). Apical and subapical polyps were identified and their growth was compared (Supplementary Fig. 3). 3D models from the beginning and the end of the experiment were compared using the Meshmixer software, to determine differences in volume and surface.

### Bacterial community sampling and DNA extraction

For each experimental condition, three polyps *per* colony of *L. pertusa* were sampled at the start of the experiment (T0), and after 2, 4, 6, and 8 weeks. Additionally, at each sampling time, one litre of aquarium seawater was sampled and filtered sequentially through a 3 µm pore-size polycarbonate filter (Millipore, Darmstadt, Germany) followed by a 0.22 µm filter. Coral samples and the filters were flash-frozen in liquid nitrogen and then stored at − 80 °C until nucleic acid extractions. For DNA extraction, individual polyps (including skeleton, tissues, and mucus) were first crushed using a sterile hammer, then ground in tubes containing a garnet matrix, and lysed mechanically using a FastPrep Instrument (MP, Biomedical, llkirch-Graffenstaden, France). DNA extraction was performed using the Maxwell Blood DNA Purification Kit LEV and the Maxwell 16 MDx Instrument (Promega, Madison, WI, United States) following the manufacturer’s instructions. The FastPrep grinding and lysing protocol was also applied for the seawater samples after cutting the 0.22 µm filters into small fragments.

### 16S rRNA amplicon sequencing and data analysis

The V1–V3 region of the bacterial 16S rRNA gene was amplified by PCR using the primers 27F-AGRGTTTGATCMTGGCTCAG [37] and 519R-GTNTTACNGCGGCKGCTG [73] with the HotStarTaq Plus Master Mix Kit (Qiagen, Valencia, CA, United States) and high-fidelity Phusion polymerase under the following conditions: 30 s at 98 °C, 16 cycles of 98 °C for 10 s, 60 °C for 30 s, 72 °C for 80 s and final extension for 5 min at 72 °C. Following the PCR, all the amplicon products from the different samples were mixed in equal concentrations and purified using Agencourt Ampure beads (Agencourt Bioscience Corporation, MA, United States). The DNA library was prepared using the purified PCR products following the Illumina TruSeq DNA library preparation protocol. All the samples were sequenced on the same Miseq Illumina sequencer run using Miseq reagent kit V3 (Illumina, CA, United States), producing 2 × 300-bp long reads. PCR and sequencing were conducted by a commercial laboratory (Integrated Microbiome Resource, Halifax, Canada). All 16S rRNA sequences were deposited in GenBank under SRA accession number PRJNA1085650.

Sequence analysis was performed with Dada2 in R [15], v 1.26.0). We applied the standard pipeline with the following parameters: trimLeft = 20, truncLen = c(290, 270), maxN = 0, maxEE = c(2,5), truncQ = 2. The sequences were filtered, dereplicated, and chimeras removed, to obtain amplicon sequence variants (ASVs). ASVs were classified against the SILVA v. 128 database [65] for taxonomic assignment. An additional BLAST [4] search was performed on the ASVs selected by SIMPER analysis with the vegan package [60].

### Metagenomic sequencing and data analysis

A total of 12 samples (two *per* temperature condition at T0 and after 8 weeks) were used for sequencing metagenomes generated with Truseq DNA Nano and sequenced on Illumina Novaseq (2 × 150 bp) with 80 Gb/sample as target. After removing adapters and quality filtering using Cutadapt, sequences from each metagenome were assembled individually with MegaHIT [44] using the *meta-large* options. Subsequently, EukRep [80] was used to identify and segregate eukaryotic from prokaryotic contigs, using a minimum contig size of 2 Kbp. Eukaryotic contigs (coral host and protistan symbionts) were then removed from the dataset. Prokaryotic genes were predicted on prokaryotic contigs using two distinct tools: MetaGeneMark, a metagenomic gene discovery tool [82], and Prodigal, a software designed for the prediction of proteins within prokaryotic genomes [32]. While MetaGeneMark could predict both complete and partial genes, Prodigal focused exclusively on complete genes. To ensure downstream analysis quality, only genes with a length of 250 bp or more were retained.

To build a gene catalog, the predicted genes from the different samples were pooled and dereplicated at 95% similarity and 80% alignment coverage with 'linclust' [70]. Metagenome reads were back-mapped to the catalog using BWA [45], and the number of counts *per* gene was obtained using HTSeq [2]. Counts *per* gene were normalized by gene length and the geometric mean abundances of 10 selected single-copy genes in each sample [67]. Normalized gene abundance tables were generated, including the abundance of each gene (ORF) in each sample. The corresponding functional abundance tables were generated by adding all the normalized abundances of all genes annotated to a specific function within a given database (e.g., KEGG). Prokaryotic genes were taxonomically annotated with MMseqs2 against the Genome Taxonomy Database (GTDB) [63], and functionally annotated using blastp with Diamond [v0.9.22] against the KEGG (Kyoto Encyclopedia of Genes and Genomes database.

All metagenomic sequences were deposited at the ENA under accession number PRJEB68224.

### Statistical analysis

Tests for normality of variance were performed using the Shapiro–Wilk test with the R software (v. 4.2.2). The distribution of survival data was normal, allowing a multiple factors ANOVA analysis. HSD post hoc tests were performed using the *TukeyHSD* function to determine differences among temperature conditions. As the distribution of growth rates was not normal (p < 0.05), a non-parametric multiple comparison Kruskal–Wallis (K–W) test was used to test possible statistical differences between thermal conditions and sampling times.

The vegan package [60] was used for the following computations. Diversity was assessed by calculating the Shannon diversity index [69]. A nMDS based on Bray–Curtis similarity was constructed using the Hellinger transformed ASV table [41]. Significant differences between community composition were tested with PERMANOVA with the *adonis* function. Homogeneity of variances was tested with the *betadisper* function followed by *permutest*. The assumption of homogeneity was respected for the comparison of the temperature conditions through time. A simper test was then performed [81] to identify the ASVs that contributed the most to the differences between temperature conditions.

DESeq2 [46] was used on the gene abundance table to identify the genes that varied the most between temperature conditions. A nMDS based on Bray–Curtis similarity was then constructed using the genes that vary the most and the significant difference between groups was tested with PERMANOVA with the *adonis* function in vegan.

## Results

### Polyp survival and skeletal growth

Survival differed significantly between temperature conditions (F_2,20_ = 106.209, p < 0.001), and across time within these conditions (F_8,20_ = 7.029, p < 0.001) (Fig. 1b). More precisely, survival rates were overall significantly lower at 15 °C, with high polyp mortality after 15 days (survival of 52 ± 17%, Tukey HSD post hoc test p < 0.001), and decreased continuously until the end of the experiment (survival of 31 ± 13% at 8 weeks, Supplementary Table 2). Survival rates also differed significantly between sampling times (F_4,20_ = 95.485, p < 0.001) after 2 weeks for 13 and 15 °C (60 ± 12% and 34 ± 13%, respectively), and after 8 weeks in the control conditions (73 ± 9%). Finally, survival rates varied significantly between colonies (F_4,20_ = 88.024, p < 0.001), with some colonies (e.g., colonies 8 and 9) displaying higher survival rates than others (Tukey HSD post hoc test p < 0.001, Supplementary Fig. 4).

The average polyp linear growth rate was not significantly different between corals exposed to water at 10 °C (2.6 ± 2.3 mm y^−1^), 13 °C (2.2 ± 1.7 mm y^−1^), and 15 °C (3.5 ± 2.9 mm y^−1^) (Kruskall–Wallis, n = 44, p > 0.05) (Supplementary Table 3). Apical (i.e., the younger polyps at the summit of the branch that drive the linear extension) and subapical polyps showed no significant differences in linear growth rates (K–W, n = 44, p > 0.05, Supplementary Table 3). No budding (i.e., formation of new polyps) was observed during the experiment.

Despite promising image quality (Supplementary Fig. 2), the low polyp growth rates were close to the detection limit of the 3D scanner apparatus (0.01 mm, manufacturer data), which thus prevented the use of biovolume and surface analyses to measure growth (Supplementary Table 4). It is therefore recommended to use corals with higher growth capabilities and/or on a longer time scale in future studies with CWCs.

### Coral microbiome

**Fig. 2:**
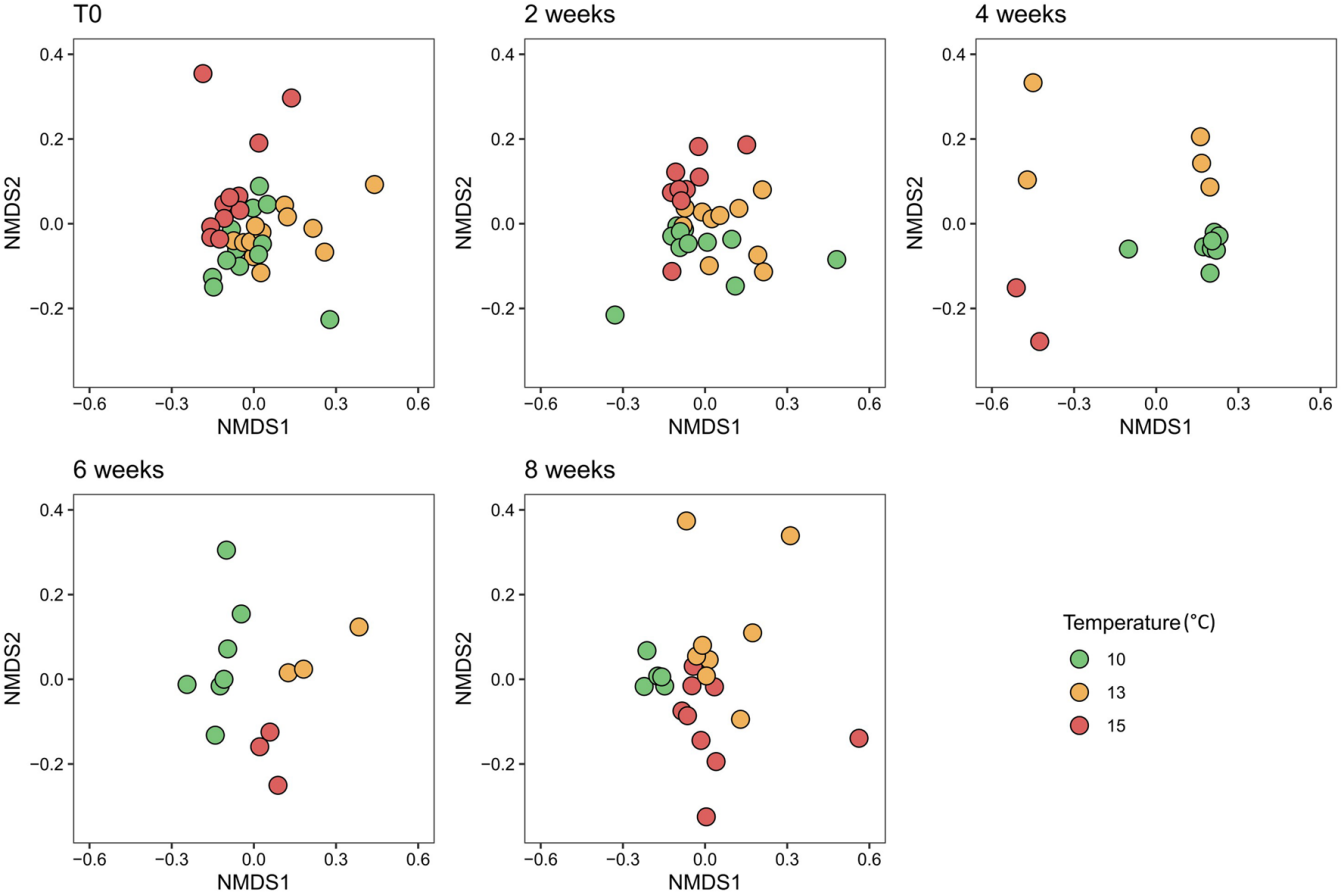
Non-metric multi-dimensional scaling plot (nMDS) based on the Bray–Curtis similarity index showing similarity between bacterial community compositions of *L. pertusa* at the start of the experiment (T0), and after two, four, six, and eight weeks at 10 °C, 13 °C, and 15 °C

The non-metric multidimensional scaling ordination (nMDS) showed that at the start of the experiment (T0), the samples from the different temperature conditions grouped together, (Fig. 2). After 2 weeks, *L. pertusa* samples at 15 °C started separating from the 10 and 13 °C samples. By 4 weeks, the samples from the different temperatures had separated from each other, and this separation was maintained after 6 weeks. After 8 weeks, the 13 and 15 °C samples were more dispersed and remained separated from the 10 °C samples, which were more clustered. A non-parametric multivariate analysis of variance (PERMANOVA) revealed significant differences between temperatures at all experimental times. However, toward the end of the experiment, higher pseudo *F-*ratios values indicated a more pronounced separation between temperature groups, and higher R^2^ showed that temperature explained variation in the model better at the end of the experiment than at the beginning (Supplementary Table 5).

The bacterial community composition of the water was different from the coral bacterial communities (Supplementary Fig. 5, PERMANOVA, F = 23.18, R = 0.15), and was similar between temperature conditions during the experiment.

**Fig. 3:**
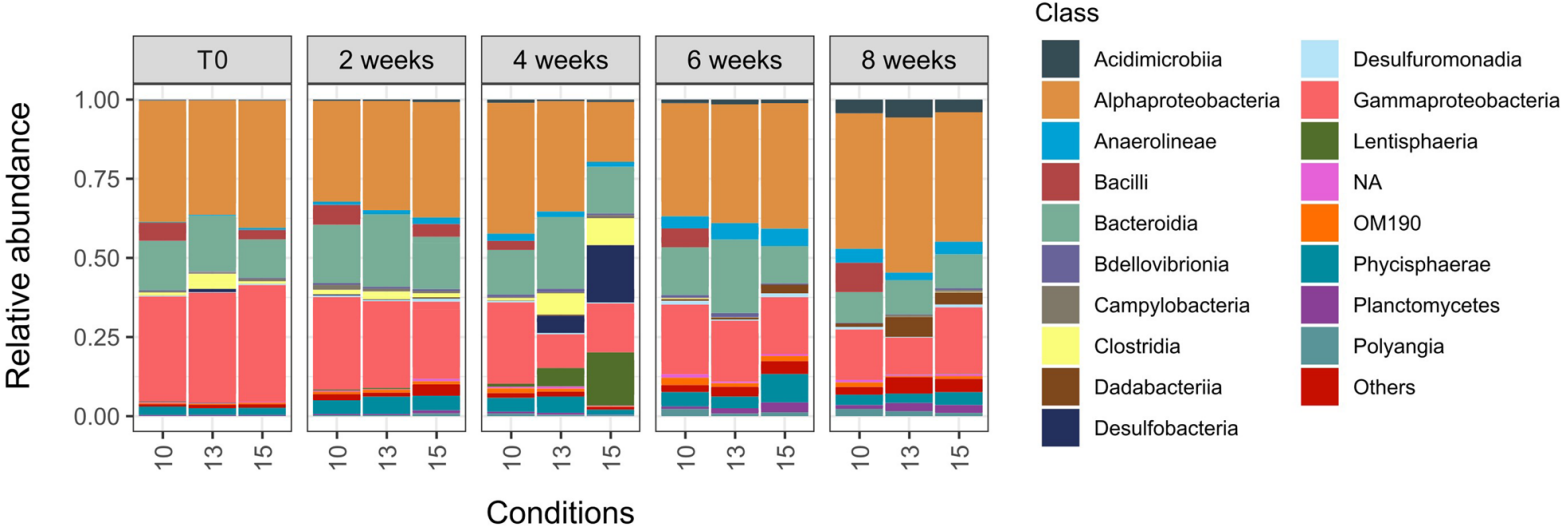
Relative abundance of bacterial sequences at the class level (18 most abundant classes) in *L. pertusa*. The composition is based on average values over the triplicate of each colony at each temperature condition (10 °C, 13 °C, and 15 °C) at the start of the experiment T0, and after two, four, six, and eight weeks of the experiment

Regarding the bacterial community composition at the class level, *L. pertusa* bacterial communities were dominated by ASVs belonging to Alphaproteobacteria, which represented respectively in average along the experiment 37.5, 38 and 34% of the sequences at 10, 13 and 15 °C (Fig. 3). Gammaproteobacteria were also one of the main classes characterizing the bacterial communities but tended to decrease slightly at the end of the experiment in all temperature conditions. Both Desulfobacteria and Clostridia had the highest abundance after 4 weeks at 13 and 15 °C (Fig. 3). Acidimicrobiia had highest relative abundance after 8 weeks.

The overall bacterial community diversity did not differ between temperature conditions (Shannon diversity index, K–W, p = 0.61), but differed through time (K–W, p < 0.01). Community diversity was consistently highest at the start of the experiment and decreased over time (Supplementary Fig. 6).

**Fig. 4:**
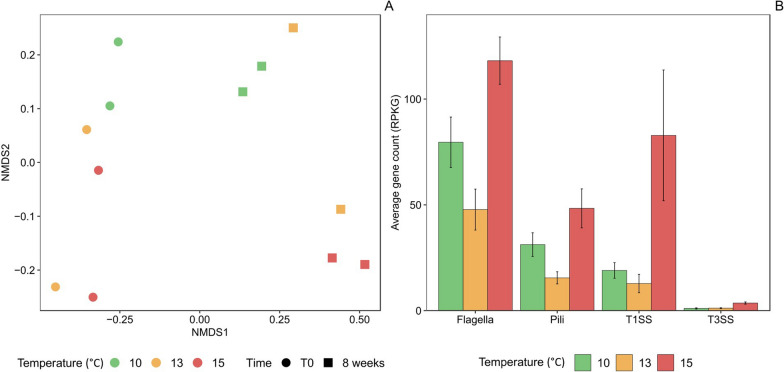
Non-metric multi-dimensional scaling plot (nMDS) based on the abundance of the functional genes that varied the most between temperature conditions (n = 5018) at the beginning and the end of the experiment as identified with DESeq2 (**a**). Abundance of genes annotated as belonging to pili and flagella formation, and involved in type 1 and 3 secretion systems (T1SS and T3SS) at the end of the experiment under incubation at 10 °C, 13 °C, and 15 °C (**b**)

Metagenomes were sequenced from samples taken at the beginning and the end of the experiment for all temperature conditions to identify bacterial genes that could vary in relative abundance between experimental conditions. The nMDS based on the genes that varied the most, identified with DESeq2, revealed that at T0, there were no significant differences in gene composition between temperatures (PERMANOVA, p = 0.14). However, after 8 weeks, the gene composition differed between coral microbiomes incubated at different temperatures (PERMANOVA, p = 0.04) (Fig. 4a). No distinct patterns of sample separation were observed considering all genes together (Supplementary Fig. 7).

We then specifically compared gene composition between 10 and 15 °C after 8 weeks of incubation with DESeq2. Among the 69 genes that varied the most in relative abundance, only 5 could be annotated, and only one showed consistent variations between replicates. This gene was annotated as coding for hemolysin A secretion system, which is involved in pathogenicity (KEGG K11004). This prompted us to search for other gene markers potentially indicative of the presence of pathogenic bacteria. We therefore targeted genes involved in type 1 and 3 secretion systems (T1SS and T3SS), as well as pili and flagella construction (Supplementary Table 6). On average, the relative abundance of genes associated to T3SS, T1SS, pili, and flagella was significantly higher in the microbiome of corals incubated at 15 °C compared to 10 °C and 13 °C (Fig. 4b, t-test, p < 0.01).

**Fig. 5:**
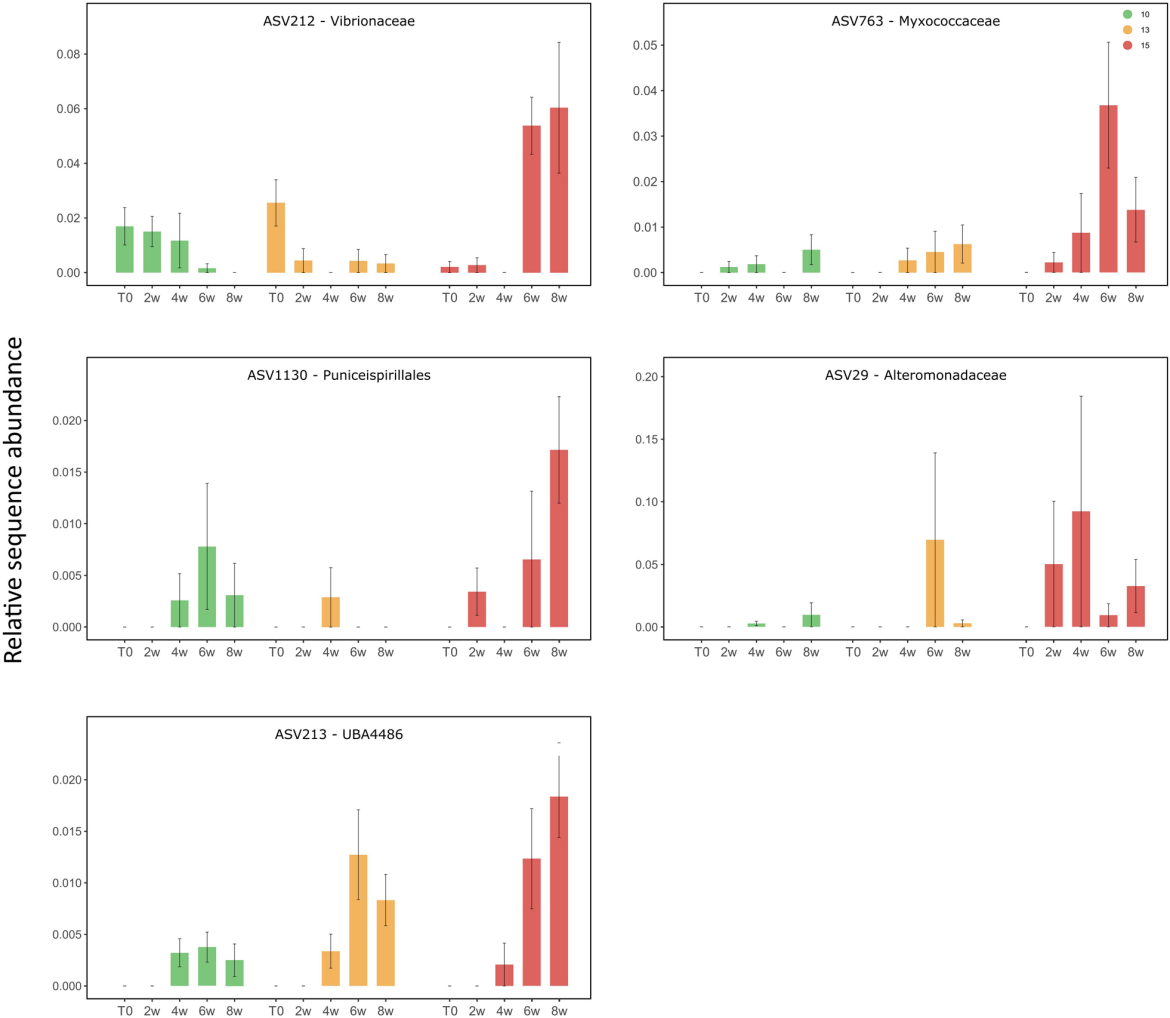
Relative sequence abundance of potential pathogens characterizing *L. pertusa* bacterial communities under different experimental conditions (10 °C, 13 °C, and 15 °C) at the start of the experiment (T0), and after 2 weeks (2w), 4 weeks (4w), 6 weeks (6w), and 8 weeks (8w) of experiment. Mean values and standard deviations are presented

The taxonomic annotation of the functional genes allowed us to find some 16S rRNA ASVs with the corresponding taxonomy. Among the genes coding for flagella, we identified two corresponding ASVs annotated at the genus level as *Vibrio* (Vibrionaceae) and uncultured *P3OB-42* (Myxococcaceae) (Fig. 5). These ASVs (ASV212 and ASV763) had highest relative abundance at 15 °C at the end of the experiment. Among genes coding for the T1SS, we identified one *Puniceispirillales* (Alphaproteobacteria) (ASV1130) that also had highest abundance after 8 weeks at 15 °C, and one *UBA4486* (Gammaproteobacteria) (ASV213) and one Alteromonadaceae (ASV29), that were abundant at both 13 °C and 15 °C at the end of the experiment (Fig. 5).

Within the 16S rRNA amplicon dataset, we identified additional ASVs that became more abundant at 15 °C. ASV150, family Saprospiraceae, and ASV446, family Moritellaceae, were not present at 10 °C and 13 °C but observed at 15 °C (Supplementary Fig. 8). Inversely, some ASVs were present at 10 °C throughout the experiment, but had lower abundance at both 13 and 15 °C by the end of the experiment (ASV20, order Alteromonadaceae and ASV65, order Rhodobacteraceae, Supplementary Fig. 8).

Finally, some colonies harboured specific ASVs that were unique to their colony (e.g., ASV235, order Thermoanaerobaculaceae, in colony L9, Supplementary Fig. 9), or were present at very low abundance in other (i.e., ASV4, family *Spiroplasmataceae*, in colony L8, Supplementary Fig. 9). Within these colonies, these ASVs persisted under different temperature conditions and throughout the different times of the experiment (Supplementary Fig. 9).

## Discussion

We demonstrate that warming had a negative impact on the *Lophelia pertusa* holobiont from the north-east Atlantic Ocean. Within 8 weeks, survival dropped from 60% at 13 °C (+ 3 °C) to 33% at 15 °C (+ 5 °C). Several lines of evidence indicate that coral mortality could be due to the action of pathogenic bacteria that proliferated within the host during the course of the experiment. Metagenomic analysis showed that bacterial gene composition differed between coral microbiomes incubated at different temperatures. Notably, several genes that were more abundant at higher temperatures were coding for secretion systems (T3SS and T1SS), pili, and flagella. Swimming motility is an important factor in bacterial colonization and infection [75]. Indeed, the flagellum is known to play a crucial role in chemotaxis and adhesion to the coral during the infection by *Vibrio* species in tropical reefs [50]. In turn, secretion systems allow the direct injection of effector proteins into the extracellular medium (T1SS), or directly into host targeted cells (T3SS), contributing to pathogen infections [7, 20]. Increased abundance of microbial genes involved in virulence, motility, and chemotaxis has previously been observed in response to stress, including increased temperature, in the tropical coral *Porites compressa* [77]. In our study, the genes potentially indicative of virulence were in particular associated with a Vibrionaceae*,* a Myxococcaceae*,* a Puniceispirillales, and a Gammaproteobacteria that were more abundant at higher temperatures. These bacteria may be part of the pathogens invading stressed corals, although these bacterial families, including Vibrionaceae, also comprise non-pathogenic members [58].

Other potential pathogens were detected by 16S rRNA metabarcoding. Several bacteria from the Saprospiraceae order were only present at 15 °C. This order has previously been detected in tissues of the tropical coral *Acropora muricata* affected by White Syndrome [71] and heat stressed *Stylophora pistillata* [68], suggesting their potential implication as opportunistic pathogens. Similarly, Clostridia earlier identified as potential pathogens in tropical coral [51] were present at 13 and 15 °C. In a meta-analysis, Mouchka et al. [56], showed that Clostridia, together with Rhodobacter and Cyanobacteria, appeared to increase in abundance in majority of diseased tropical corals. Concurrently, they observed that bleached corals had a higher proportion of opportunist bacteria such as *Vibrio* sp. than healthy colonies. Similarly, Vega Thurber et al. [77] showed that thermally stressed tropical corals exhibited specific disease-associated microbiome, with a low abundance of *Vibrio* sp., and distinct microbiome metabolisms and functioning. Our results reveal for the first time that stressed cold-water corals display microbiome shifts toward a higher proportion of opportunistic or potentially pathogenic bacterial taxa in the same way as tropical corals. It remains, however, to be demonstrated if these bacteria were already present in very low abundance in or on the coral at the start of the experiment, or if they originated from the surrounding water [43]. Changes in bacterial community composition (dysbiosis) were due to the appearance of higher abundance of opportunistic and potentially pathogenetic bacteria, as detailed above, but also to the concomitant disappearance of bacteria present in control conditions. These changes appeared early in the experiment (after 2 weeks), which suggests a rapid stress-induced dysbiosis under warming conditions. Altogether, our results reflect a limited capacity of the coral to maintain or regulate its microbiome under elevated temperature, which results in a proliferation of potentially pathogenic bacteria, especially for a 5 °C increase.

Mortality under elevated temperature has been reported earlier in *L. pertusa* from different regions during short- and long-term experiments, and 14–15 °C is generally considered the upper limit of thermal tolerance for this species [10]. Previous experiments showed that Mediterranean *L. pertusa*, normally living at 13 °C, were strongly affected by water temperatures of 17 °C, with only 50% survival after 2 months of exposure, and only 20% after 6 months, whereas no mortality occurred at 15 °C [19]. In the Gulf of Mexico, where corals live between 7.0 and 9.5 °C, Lunden et al. [47] reported 54% and 0% of survival after 15 days at 14 °C and 16 °C respectively, while Brooke et al. [10] reported complete mortality of corals at 25 °C after 24 h of exposure, and 80% of survival after 7 days at 15 °C. In light of our results and the variations observed in earlier studies, we suggest that the level of temperature increase relative to the natural conditions (e.g., + 5 °C), rather than a fixed arbitrary value (e.g., 15 °C), should be considered when predicting coral survival in different habitats. We hypothesize that *L. pertusa*, wherever they are from, can only survive a temperature increase < 3 °C over a long period. Regional variations in deep-sea water temperature increase should therefore be considered before estimating the future global distributions of cold-water corals.

We observed differences in survival between colonies, suggesting intra-species variability with the probable presence of genotypes that are more sensitive or more resilient to environmental changes. Interestingly, we also observed colony-specific differences at the microbiome level. Some colonies had unique bacteria that were almost or totally absent in others (e.g., *Spiroplasmataceae* (class Mollicutes) and *Thermoanaerobaculaceae*). Variation between colonies of *L. pertusa* have been previously documented for different physiological parameters [13, 24, 28, 31, 36, 47], including for their microbiome [34, 35, 48]. The *Thermoanaerobaculaceae* found in colony L9 had only 97% similarity to the closest hit in the databases (a sequence found in the sponge *Halicona tubifera* [23], and the *Spiroplasmataceae* from colony L8 had only 91% similarity to reference sequences, so we could not directly relate our data to the existing literature. Interestingly, the colony L8 harboring the *Spiroplasmataceae* exhibited one of the highest survival rates at 10 °C. Although our experimental design did not allow us to infer a direct relationship between colony-specific microbiome and survival, we can hypothesize that the microbiome could play a role. Future investigations should consider the microbiome when exploring inter-individual variations and their possible role in the resilience of specific genotypes within a reef or a population [79]. It is of paramount importance for predicting potential population adaptation within the context of global change.

We report here the first growth rate estimations for *L. pertusa* from the Bay of Biscay, and we observed that they are in the lower range compared to values published for this species in other geographical areas, both in aquaria and in situ. In situ measurements showed *L. pertusa* growth rates ranging from 2.44 to 32 mm y^−1^ in the Gulf of Mexico [11, 38], 1–40 mm y^−1^ in the Mediterranean Sea [18, 40], from 1 to 26 mm y^−1^ in Norway [14, 53] and up to 26 mm y^−1^ in the North Sea [27]. Growth rates are usually lower in aquaria experiments where they range from 1 to 17 mm y^−1^ for Mediterranean *L. pertusa* [39, 61], and are up to 9.4 mm y^−1^ for corals from Norway [57]. The values measured in our study never reach these maxima. However, considering that no budding (i.e., new polyp formation) occurred during the 2 month experiment, and that the growth rates of old polyps is significantly lower than that of new ones [39], it is not surprising to observe such low values in aquaria. The growth rates measured here are close to those found by Chapron et al. [19] using a similar experimental setup. Earlier studies conducted in aquaria collected corals originating from shallow depths in Norwegian fjords [62], to 690 m depth in the Mediterranean Sea at the deepest [39, 59]. Here, the specimens collected in the Lampaul canyon were from a depth of 800 m, which, to our knowledge, corresponds to the deepest corals maintained in aquaria for such medium-term experiments. The lower growth rate may, therefore, also be explained by the fact that our corals originated from deeper waters, and since maintaining such a deep population in aquaria at atmospheric pressure could be detrimental to their health. This hypothesis is supported by the lack of difference in growth rates between subapical and apical polyps, which normally grow faster [19]. The mortality observed in the control conditions (72% survival at 10 °C after 2 months), despite stable physico-chemical conditions, further indicates that aquarium conditions may not be optimal for these deep corals, which were collected at 800 m depth. Alternatively, the lower growth rate may simply reflect the different ecological properties of the Lampaul canyon corals. A better characterization of in situ coral biology is thus required.

In the present study, temperature had no significant effect on skeletal growth rates, which contrasts with results from previous studies. Based on similar temperature values for Mediterranean *L. pertusa* (i.e., 10, 13 and 15 °C), Chapron et al. [19] observed the highest growth rates at 13 °C, in the in situ conditions, and a lower at both 10 and 15 °C. A decrease of calcification rates with lower temperatures was described by Naumann et al. [59] on Mediterranean *L. pertusa* when exposed to 12 °C and 6 °C, but these corals were placed in lower temperatures compared to in situ conditions (i.e., ~ 13 °C at 300 m depth in the Cap de Creus canyon, [74]). A warming experiment on *L. pertusa* from a Norwegian fjord revealed higher calcification rates for corals exposed to 12 °C compared to 8 °C, their natural habitat conditions [13]. This suggests that corals from different regions may respond differently to a changing environment and exhibit varying levels of sensitivity. While an increase in growth rates is not necessarily indicative of good health status, the lack of temperature impact on polyp growth rates during our two-month experiment indicates that corals had likely maintained sufficient reserves and/or metabolism that could be invested to sustain growth. However, as cold-water corals are known to be slow-growing species compared to tropical corals, a longer-term experiment could have allowed to detect more subtle differences in growth response [19, 55].

## Conclusion

We showed that an increase in water temperature of + 3 °C and + 5 °C was responsible for dramatic mortality in *L. pertusa*. Mortality appears to be associated with bacterial pathogens, as evidenced by the substantial increase in the number of genes coding for virulence factors such as motility and secretion systems. In more resilient individuals, while the polyps survived, elevated temperatures led to rapid changes in the associated bacterial community composition (dysbiosis). Interestingly, some specific colonies harboured specific microbiomes, suggesting that colony-specific traits contribute to varying resilience to environmental changes. Finally, our results suggest that NE Atlantic *L. pertusa* are as sensitive to warming as Mediterranean or Gulf of Mexico populations. Although the NE Atlantic *L. pertusa* have a lower upper thermal limit than other *L. pertusa* populations (< 13 °C *vs* < 15 °C), it appears that all *L. pertusa*, regardless of the region they originate from, and the water temperature in which they live, will be strongly impacted by a + 3 °C increase. Future works on the thermal tolerance of cold-water corals should, therefore, consider the level of temperature increase in an ecological context.

## Supplementary Information

Additional file 1

## Data Availability

Samples and their metadata were registered in the ENA and Genbank biosample database. All sequencing files were submitted to the European Nucleotide Archive (ENA) at the EMBL European Bioinformatics Institute (EMBL-EBI) and GenBank under accession number PRJEB68224 (metagenomic sequences) and PRJNA1085650 (metabarcoding sequences) respectively.
