# Supplementary material for: Cold-water coral mortality under ocean warming is associated with pathogenic bacteria
Source: Environ Microbiome. 2024 Oct 16;19:76. doi: 10.1186/s40793-024-00622-0 (PMC11481251; doi:10.1186/s40793-024-00622-0)
Supplement: Supplementary file 1 — Additional file 1 [file 40793_2024_622_MOESM1_ESM.docx]

**Supplementary Table 1.** Mean (mean ± SD) physico-chemical parameters of the seawater over 8 weeks under different experimental conditions (10 °C, 13 °C and 15 °C).

| Condition | Temperature (°C) | pH | Salinity (psu) | Oxygen (% sat.) |
| --- | --- | --- | --- | --- |
| 10 °C | 10.1 ± 0.2 | 8.16 ± 0.05 | 37.9 ± 0.5 | 96.4 ± 3.3 |
| 13 °C | 12.9 ± 0.2 | 8.17 ± 0.05 | 37.9 ± 0.5 | 96.2 ± 3.2 |
| 15 °C | 14.9 ± 0.2 | 8.20 ± 0.07 | 37.8 ± 0.6 | 97.4 ± 3.1 |

**Supplementary Table 2.** Number of polyps present during the experiment at 10, 13 and 15°C at T0 and after 2, 4, 6 and 8 weeks of experiment.

| Time | 10°C | 13°C | 15°C |
| --- | --- | --- | --- |
| T0 | 120 | 122 | 124 |
| 2 weeks | 100 | 94 | 64 |
| 4 weeks | 93 | 85 | 48 |
| 6 weeks | 88 | 73 | 45 |
| 8 weeks | 87 | 72 | 42 |

**Supplementary Table 3.** Average polyp linear growth rates ± s.d. (mm yr^−1^) of *L. pertusa* for all, apical and subapical polyps, under different temperature conditions after 8 weeks of experiment. The numbers in brackets represent the number of replicates.

|  | all polyps (mm y^-1^) | apical polyps (mm y^-1^) | subapical polyps (mm y^-1^) |
| --- | --- | --- | --- |
| all | 2.9 ± 2.5 (44) | 2.5 ± 1.9 (9) | 3.0 ± 2.7 (35) |
| 10 °C | 2.6 ± 2.3 (12) | 1.2 ± 0.0 (2) | 2.8 ± 2.4 (10) |
| 13 °C | 2.2 ± 1.7 (11) | 2.5 ± 0.0 (1) | 2.2 ± 1.8 (10) |
| 15 °C | 3.5 ± 2.9 (21) | 3.0 ± 2.2 (6) | 3.7 ± 3.2 (15) |

**Supplementary Table 4.** Biovolumes (mm^3^) and surface (mm^2^) of coral nubbins obtained by 3D scanner at the start of the experiment (T0) and after 8 weeks (T4), and the calculated growth in biovolume and surface.

| Analyzed nubbin | Colony | Temperature | Scan biovolume T0 (mm^3^) | Scan biovolume T4 (mm^3^) | Growth in biovolume (mm^3^) | Scan surface T0 (mm^2^) | Scan surface T4 (mm^2^) | Growth in surface (mm^2^) |
| --- | --- | --- | --- | --- | --- | --- | --- | --- |
| L5_C10_1 | L5 | 10 | Unusable scan | 3097.44 | / | Unusable scan | 3153.9 | / |
| L6_C10_1 | L6 | 10 | 435.59 | 427.496 | -8.094 | 872.041 | 1198.48 | 326.439 |
| L6_C10_2 | L6 | 10 | 232.307 | 226.794 | -5.513 | 495.062 | 789.429 | 294.367 |
| L7_C10_1 | L7 | 10 | 1301.99 | 1137.29 | -164.7 | 1701.3 | 1856.36 | 155.06 |
| L8_C10_1 | L8 | 10 | 1242.86 | 1187.94 | -54.92 | 2306.7 | 1655.74 | -650.96 |
| L8_C10_2 | L8 | 10 | Unusable scan | Unusable scan | / | Unusable scan | Unusable scan | / |
| L5_C13_1 | L5 | 13 | 596.595 | 509.332 | -87.263 | 806.664 | 912.827 | 106.163 |
| L5_C13_2 | L5 | 13 | 1406.36 | 1274.08 | -132.28 | 2152.07 | 3267.67 | 1115.6 |
| L6_C13_1 | L6 | 13 | 750.804 | 760.317 | 9.513 | 1001.04 | 1046.28 | 45.24 |
| L6_C13_2 | L6 | 13 | 804.916 | 760.804 | -44.112 | 972.527 | 1000.49 | 27.963 |
| L7_C13_1 | L7 | 13 | 974.569 | 865.162 | -109.407 | 1166.31 | 1189.25 | 22.94 |
| L7_C13_2 | L7 | 13 | 587.249 | 593.805 | 6.556 | 863.841 | 958.735 | 94.894 |
| L9_C13_1 | L9 | 13 | 1073.19 | 1107.9 | 34.71 | 1321.01 | 1337.54 | 16.53 |
| L5_C15_1 | L5 | 15 | 1044.78 | 966.255 | -78.525 | 1486.05 | 1608.76 | 122.71 |
| L5_C15_2 | L5 | 15 | 1344.75 | 1259.4 | -85.35 | 1300.63 | 1493.75 | 193.12 |
| L7_C15_1 | L7 | 15 | 1318.9 | 1488.13 | 169.23 | 2061.5 | 1857.44 | -204.06 |
| L7_C15_2 | L7 | 15 | 1966.33 | Unusable scan | / | 3279.25 | Unusable scan | / |
| L8_C15_1 | L8 | 15 | 1103.86 | 1028.35 | -75.51 | 1574.44 | 1617.65 | 43.21 |
| L8_C15_2 | L8 | 15 | 718.696 | 661.919 | -56.777 | 990.956 | 1110.2 | 119.244 |
| L9_C15_1 | L9 | 15 | 1183.17 | 1072.79 | -110.38 | 1433.94 | 1483.06 | 49.12 |
| L9_C15_2 | L9 | 15 | 883.06 | 788.079 | -94.981 | 1330.94 | 2088.23 | 757.29 |

**Supplementary Table 5.** Summary statistics of the pairwise PERMANOVA testing the effect of temperature and time on microbial community composition.

| Time | Comparisons | R | F | p-value |
| --- | --- | --- | --- | --- |
| T0 | 10 *vs* 13 | 0.100 | 2.450 | **0.001** |
|  | 10 *vs* 15 | 0.119 | 2.826 | **0.001** |
|  | 13 *vs* 15 | 0.155 | 3.853 | **0.001** |
| 2 weeks | 10 *vs* 13 | 0.063 | 1.414 | 0.084 |
|  | 10 *vs* 15 | 0.126 | 2.601 | **0.001** |
|  | 13 *vs* 15 | 0.149 | 2.973 | **0.002** |
| 4 weeks | 10 *vs* 13 | 0.223 | 3.223 | **0.005** |
|  | 10 *vs* 15 | 0.447 | 6.460 | **0.026** |
|  | 13 *vs* 15 | 0.288 | 2.020 | 0.097 |
| 6 weeks | 10 *vs* 13 | 0.254 | 2.717 | **0.013** |
|  | 10 *vs* 15 | 0.244 | 2.582 | **0.010** |
|  | 13 *vs* 15 | 0.406 | 2.738 | 0.100 |
| 8 weeks | 10 *vs* 13 | 0.229 | 3.273 | **0.003** |
|  | 10 *vs* 15 | 0.260 | 3.162 | **0.003** |
|  | 13 *vs* 15 | 0.148 | 2.098 | **0.002** |

**Supplementary Table 6.** List of genes encoding type 1 and 3 secretion systems (T1SS and T3SS), and flagella and pili construction in the microbiome of the corals incubated at 10°C, 13°C, and 15°C.

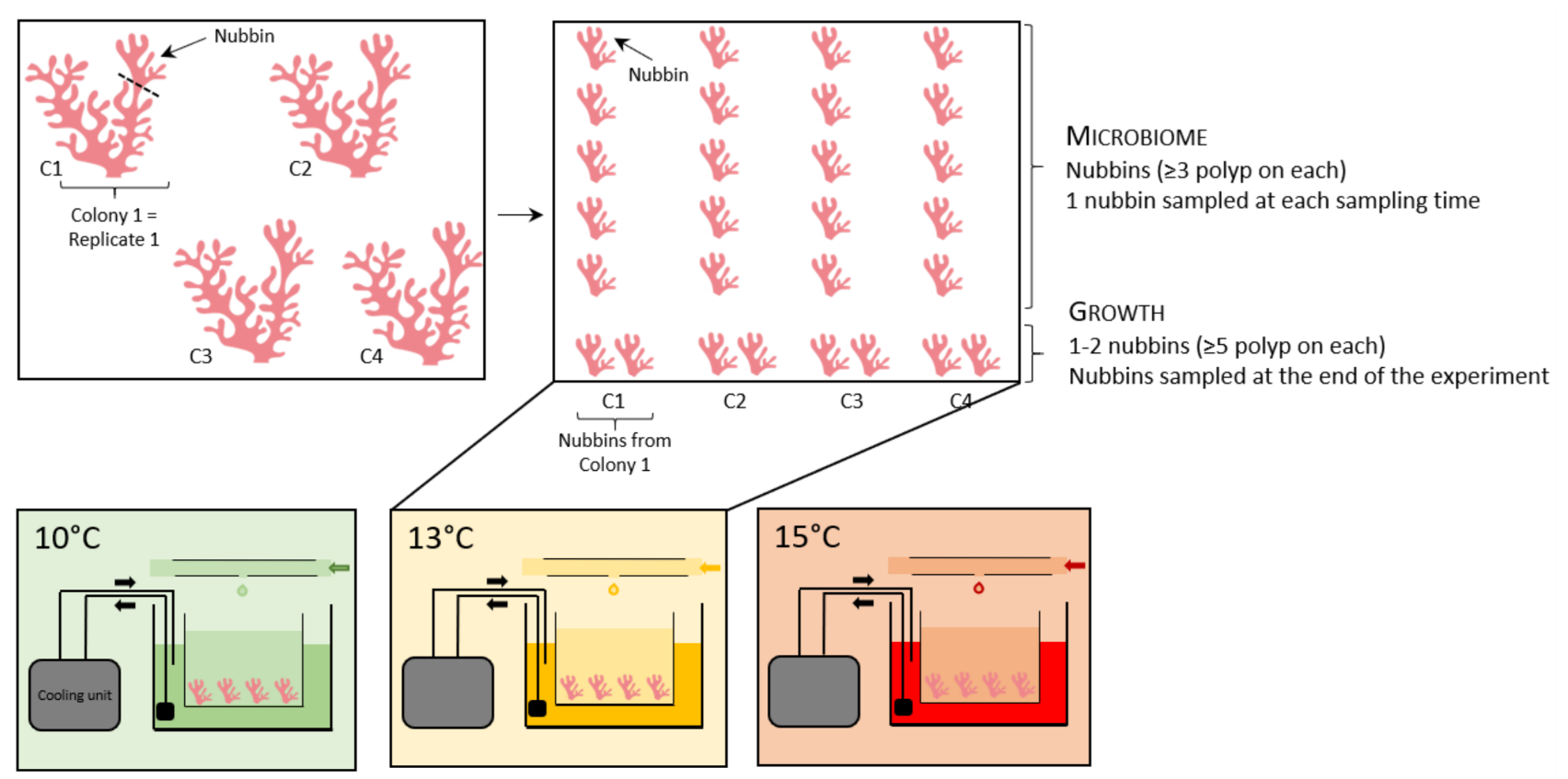


**Supplementary Figure 1.** Schematic representation of the experimental design. The upper part of the figure shows how the colonies were cut into nubbins.


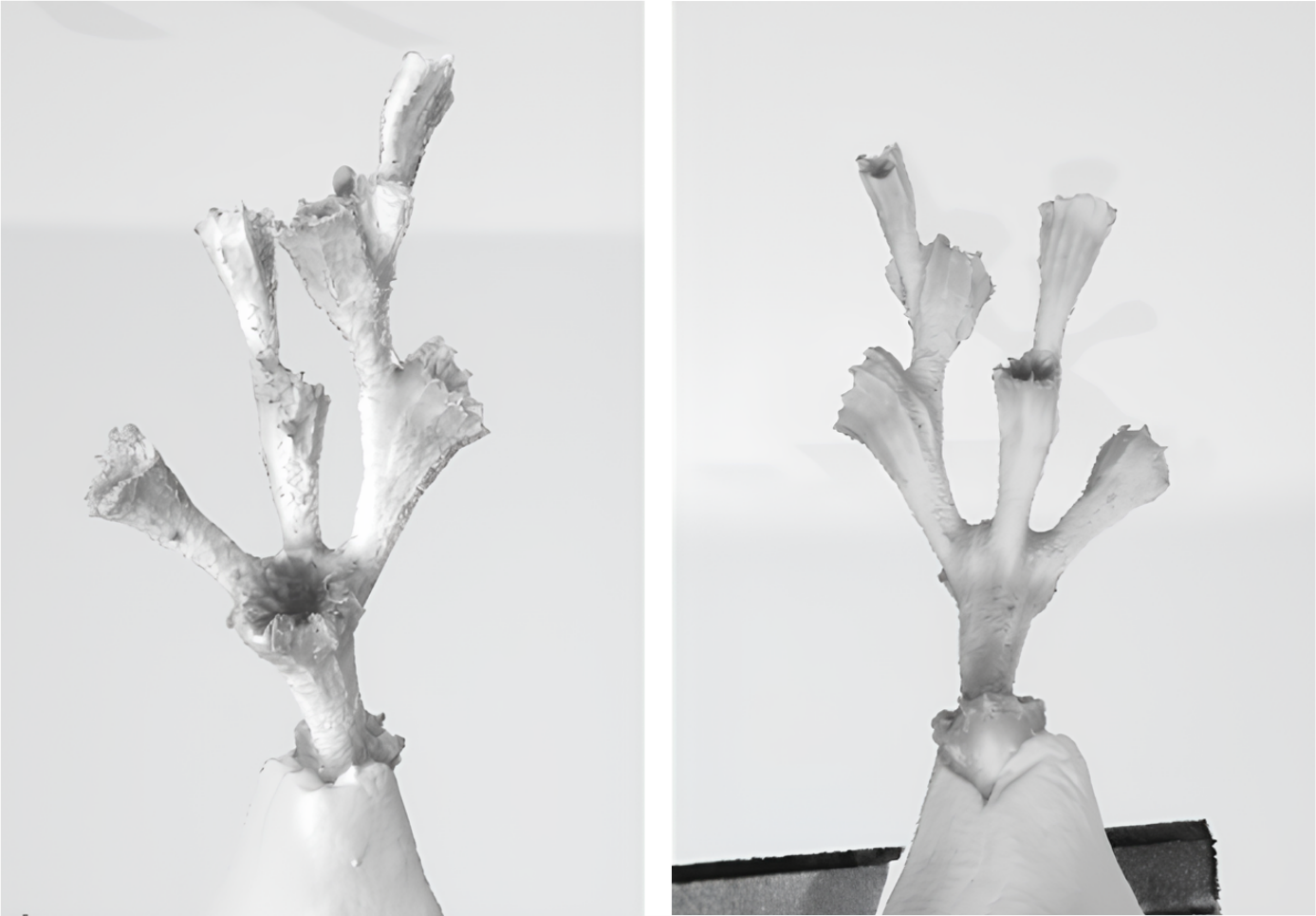


**Supplementary Figure 2.** Pictures of a scanned nubbin used for growth measurement, using AutoScan Inspec 3D scanner.


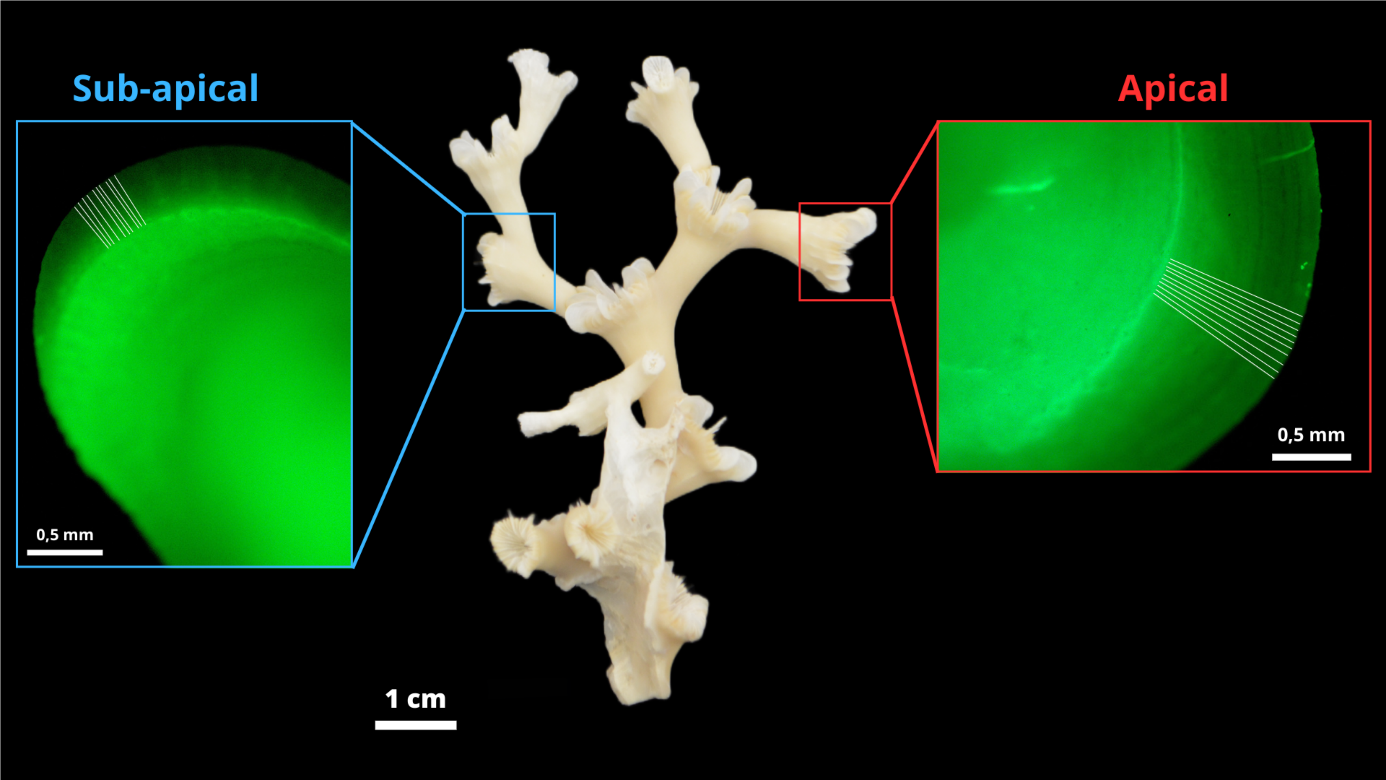


**Supplementary Figure 3.** Growth measurements (white lines) performed on *L. pertusa* septae of apical and subapical polyps under fluorescent microscope revealing the calcein staining (thickest green line).


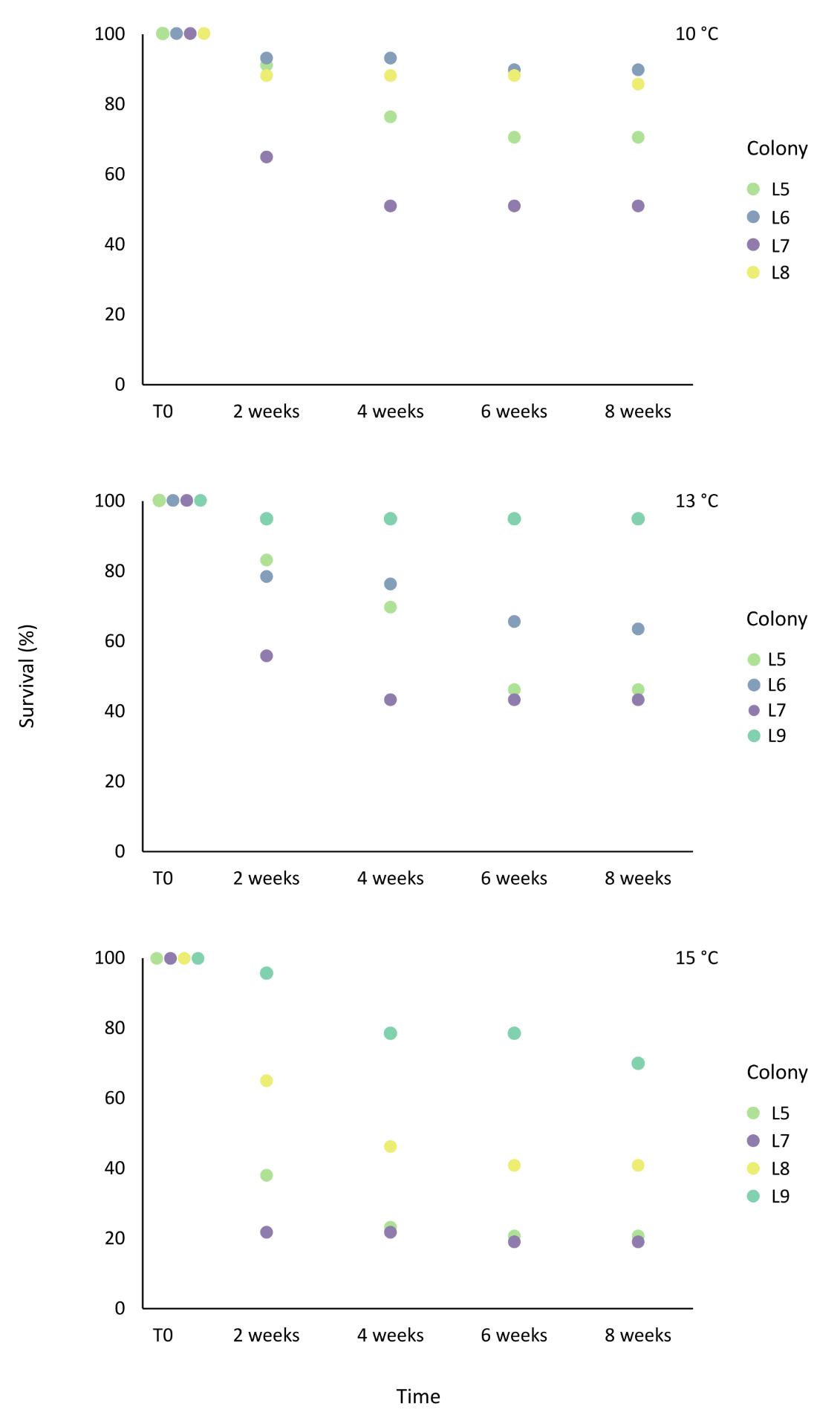


**Supplementary Figure 4.** Polyp survival rate (%) of *L. pertusa* for each colony in each temperature condition (10°C, 13°C and 15°C) during eight weeks of experiment.


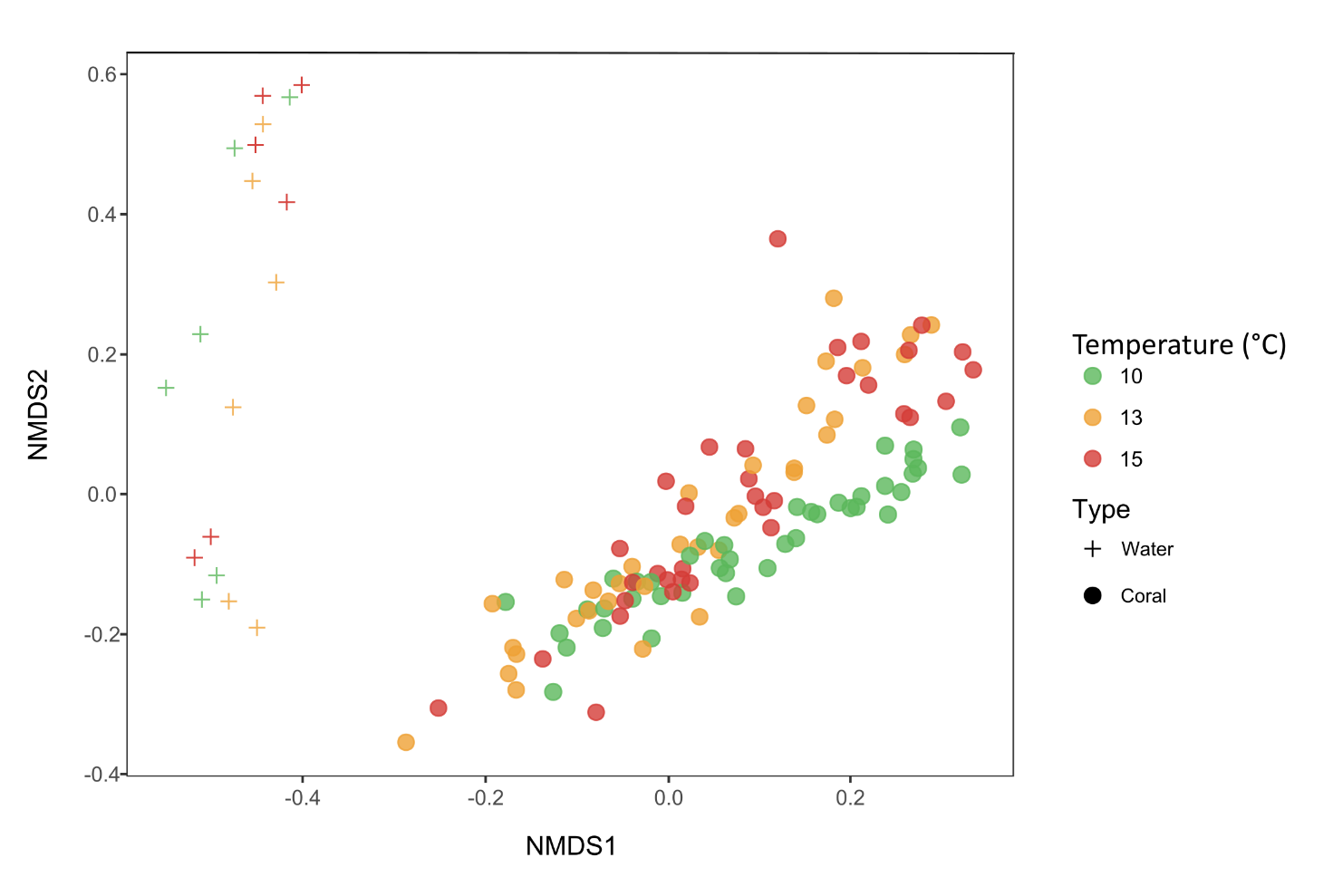


**Supplementary Figure 5.** Multi-dimensional scaling plot (NMDS) based on the Bray-Curtis similarity index showing the similarity between bacterial community compositions of L. pertusa and seawater during eight weeks of experiment at 10°C, 13°C and 15°C.


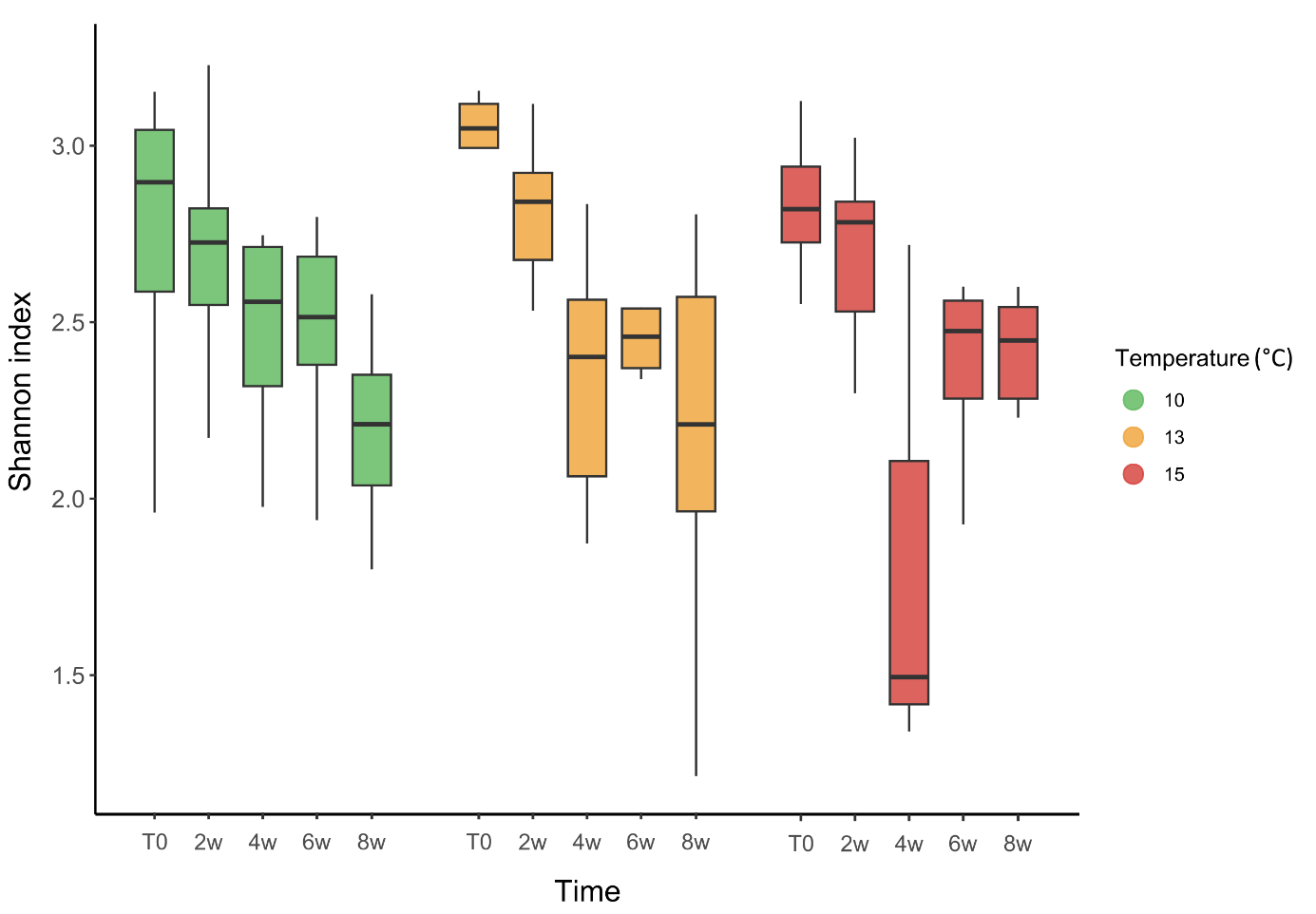


**Supplementary Figure 6.** Shannon diversity index of *L. pertusa* bacterial communities in each experimental condition (10°C, 13°C and 15°C) at the start of the experiment (T0) and after 2 weeks (2w), 4 weeks (4w), 6 weeks (6w) and 8 weeks (8w). Values presented in the box plot correspond to the median and quartile.


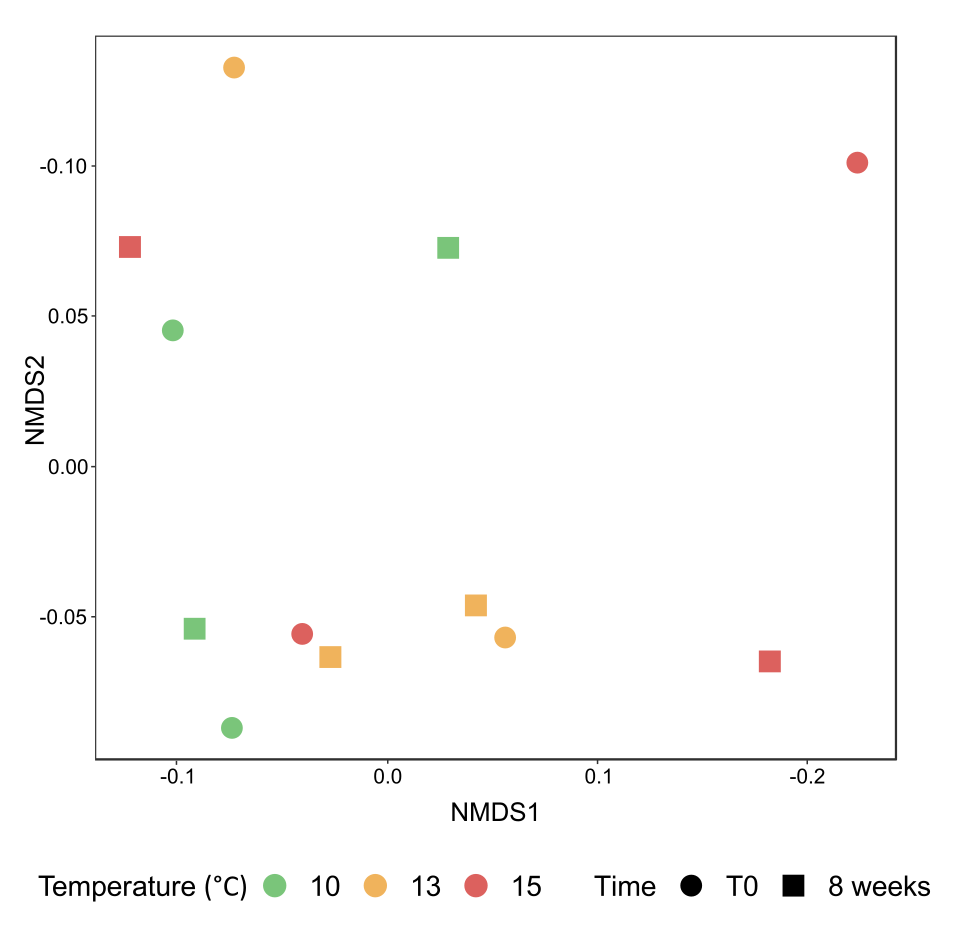


**Supplementary Figure 7.** Multi-dimensional scaling plot (NMDS) based on the relative abundance of all bacterial genes at the beginning of the experiment (T0) and after 8 weeks for corals incubated at 10^°^C, 13^°^C and 15^°^C.


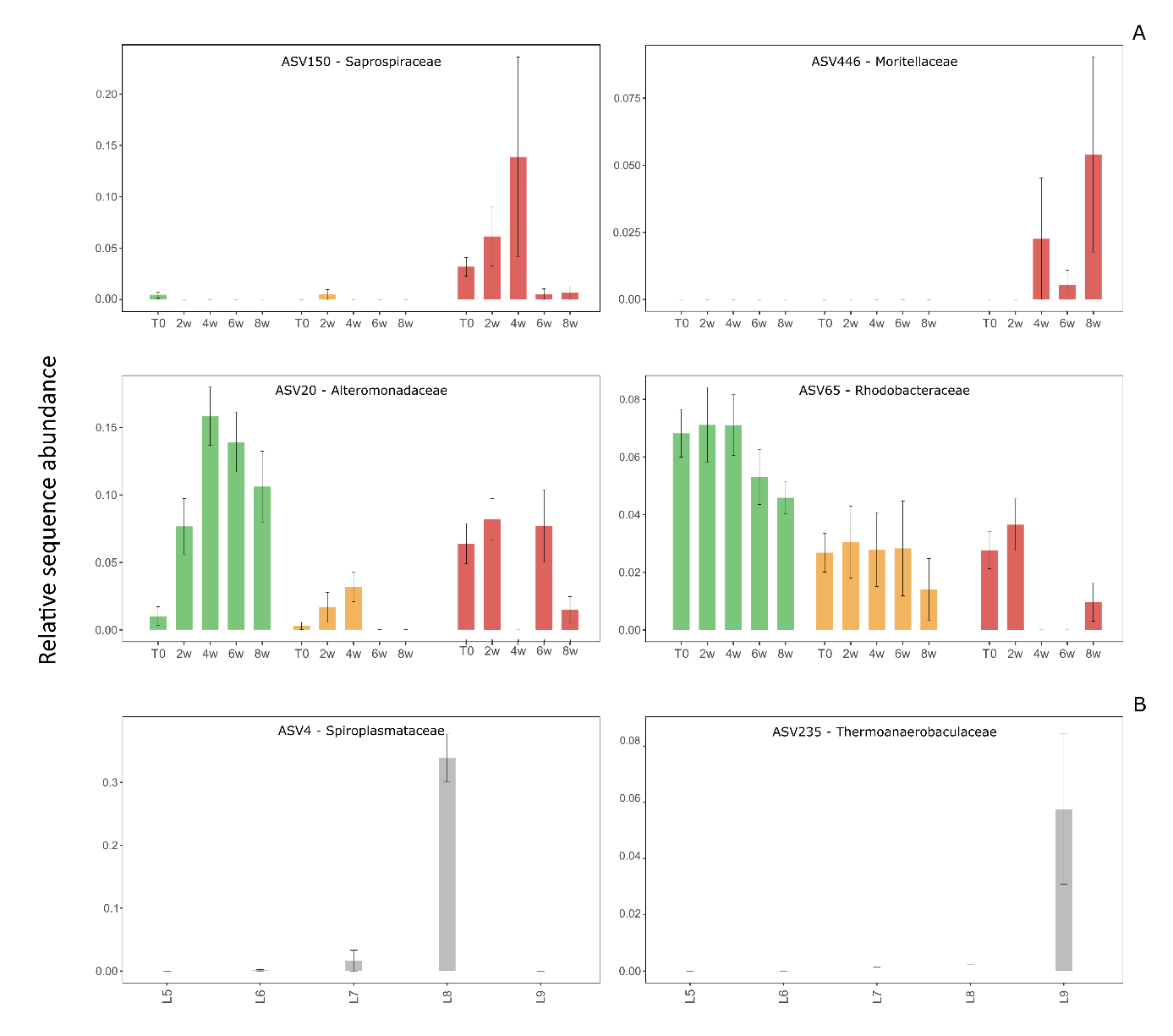


**Supplementary Figure 8.** Relative sequence abundance of ASVs characterizing *L. pertusa* bacterial communities under different experimental conditions (10°C, 13°C and 15°C) at the start of the experiment (T0), and after 2 weeks (2w), 4 weeks (4w), 6 weeks (6w) and 8 weeks (8w) (A), and unique ASVs that characterize specific colonies of *L. pertusa* during the experiment (B). The affiliation of the ASVs at the family level is given, except for ASV4 for which the affiliation at the phylum level is available. Mean values and standard deviations are presented.


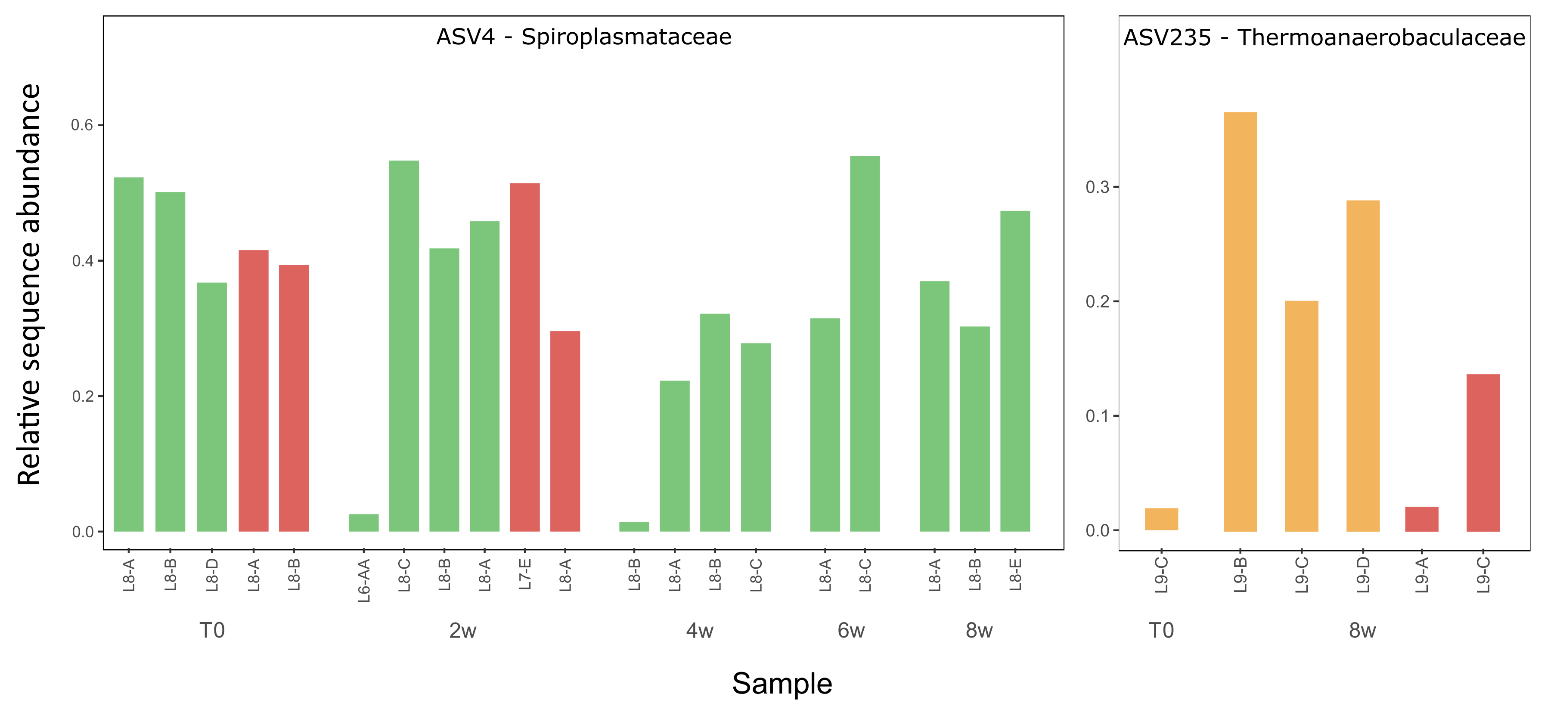


**Supplementary Figure 9.** Detailed abundance of specific ASVs that characterize particular colony of *L. pertusa* in the different temperature conditions (10°C, 13°C and 15°C) at the start of the experiment (T0), and after 2 weeks (2w), 4 weeks (4w), 6 weeks (6w) and 8 weeks (8w) of experiment.
